# Clinical Outcomes of Angiotensin II Therapy in Vasoplegic Shock: A Systematic Review and Meta-Analysis

**DOI:** 10.3390/life14091085

**Published:** 2024-08-29

**Authors:** Ans Alamami, Alaa Rahhal, Bara Alqudah, Ahmed Shebani, Abdelkarim Alammora, Hashim Mohammad, Amr S. Omar, Ahmed Labib Shehatta

**Affiliations:** 1 Medical Intensive Care, Department of Medicine, Hamad Medical Corporation, Doha 3050, Qatar; balqudah1@hamad.qa (B.A.); ashebani@hamad.qa (A.S.); ashehatta@hamad.qa (A.L.S.); 2 Pharmacy Department, Heart Hospital, Hamad Medical Corporation, Doha 3050, Qatar; arahhal1@hamad.qa; 3 Department of Medicine, Hamad Medical Corporation, Doha 3050, Qatar; aalammora@hamad.qa (A.A.); hmohammad3@hamad.qa (H.M.); 4 Cardiac ICU, Department of Cardiothoracic Surgery, Heart Hospital, Hamad Medical Corporation, Doha 3050, Qatar; a_s_omar@yahoo.com

**Keywords:** vasoplegia, shock, distributive shock, angiotensin II

## Abstract

Background: Angiotensin II is a peptide hormone vasopressor that activates angiotensin type 1 (AT1) receptors leading to vasoconstriction, the augmentation of arterial blood pressure (ABP), and organ perfusion. Angiotensin II was found to increase the ABP in catecholamine-refractory vasodilatory shock. Whether this effect improves the chances of survival or not remains inconclusive. Therefore, we conducted a systematic review and meta-analysis to evaluate the efficacy and safety of angiotensin II in vasoplegic shock. Objectives: To evaluate the clinical significance of angiotensin II effects in vasoplegic shock concerning the hemodynamic impact, mortality outcomes, and side effects. Methods: Following PRISMA guidelines, we searched PubMed and EMBASE for experimental and observational studies published in English exploring the clinical outcomes of angiotensin II use in vasodilatory shock till 1 July 2024. Two independent authors assessed the quality and risk of bias of the included studies. A random effect model (Mantel–Haenszel) was used to combine data. The primary outcome was in-hospital mortality associated with angiotensin II use in comparison to standard therapy, while the secondary outcomes were mean arterial pressure (MAP) change, multi-organ failure (MOF), and the incidence of atrial fibrillation (AF). The Q test and I^2^ were used to examine heterogeneity, with I^2^ > 50% indicating marked heterogeneity. Results: A total of eight studies (*n* = 974) comparing angiotensin II to standard therapy in vasoplegic shock were included in the systematic review, with three studies comprising 461 patients included in the final analysis of the primary outcome. Only one study evaluated the use of angiotensin II as a primary vasopressor, while the rest reported angiotensin II use in catecholamine-refractory vasodilatory shock. Overall, angiotensin II use was associated with similar in-hospital mortality compared to standard therapy (risk ratio [RR] = 0.83; 95% CI, 0.68–1.02, I^2^ = 0%). Likewise, there was no difference in MOF and AF (MOF: RR = 1.01; 95% CI, 0.61–1.65, I^2^ = 0%; AF: RR = 1.27; 95% CI, 0.38–4.23, I^2^ = 5%). However, angiotensin II use demonstrated a significant MAP increase (mean difference = −9.60; 95% CI, −9.71, −9.49, I^2^ = 0%). Conclusions: In vasodilatory shock, angiotensin II use demonstrated comparable in-hospital mortality compared to standard therapy. Nevertheless, it resulted in significant MAP change, which may encourage clinicians to use it in cases of profound hypotension.

## 1. Introduction

Vasoplegia, a clinical state characterized by abnormally low systemic vascular resistance and hypotension, is the cornerstone of the pathophysiology of refractory distributive shock. It entails a hypoperfusion state and loss of normal vascular tone, both of which, if left untreated, can have catastrophic consequences and result in multi-organ failure [1,2].

The development of vasoplegia, which is most common in sepsis and after cardiac surgery, can be ascribed to several risk factors, including the administration of angiotensin receptor blockers, calcium channel blockers, and beta blockers. The development of vasoplegia involves numerous levels of factor activation such as cytokines, platelets, leukocytes, excess nitric oxide synthesis, vasopressin deficit, and endothelial dysfunction [3,4]. The hallmarks of distributive shock management are fluid resuscitation and the administration of vasopressor agents [5,6,7]. Commonly used vasopressors are norepinephrine (NE), phenylephrine, and adrenaline, which act through the activation of adrenergic receptors. Vasopressin, a different class vasopressor, mediates its effect via the activation of the vasopressin receptor and the antagonist of nitric oxide action. Additionally, angiotensin II, a peptide hormone vasopressor produced by the liver, acts by activating angiotensin type 1 (AT1) receptors on the vascular smooth muscles leading to smooth muscle contraction and vasoconstriction in an attempt to increase the blood pressure and hold the perfusion function intact [8].

A randomized controlled trial, ATHOS-3, recruited 321 patients and sought to evaluate the therapeutic effect of angiotensin II. Around 70% of the patients assigned to the angiotensin II arm were able to reach the primary outcome of change in mean arterial pressure (MAP) of at least 10 mmHg above the baseline of more than 75 mmg. However, there was no statistically significant difference in mortality between the two groups, as the study lacked the means to detect differences in mortality [9]. Therefore, the angiotensin II impact on mortality remains uncertain.

Here we present a systematic review and meta-analysis of the clinical outcomes of angiotensin II administration in vasodilatory shock in terms of mortality, MAP change, and side effects.

## 2. Materials and Methods

We performed a systematic review and meta-analysis adhering to the Preferred Reporting Items for Systematic Reviews and Meta-Analyses (PRISMA) (Supplementary Materials) guidelines as previously described to select and appraise studies, and combine outcomes to present transparent results [10,11]. Our literature search included all experimental and observational studies assessing the clinical outcomes of angiotensin II use in vasodilatory shock published in English until 1 July 2024. Articles were excluded in case of (1) study design other than randomized controlled trials, retrospective, or prospective observational studies; (2) animal-based studies; (3) non-clinical outcomes exclusively reported; (4) interventions other than angiotensin II; (5) intra-operative use of angiotensin II; and (6) materials published in languages other than English.

We used three databases to conduct our literature search, including PubMed, Embase, and Cochrane Database, using the following terms in combination: “angiotensin II”, or “angiotensin 2”, or “ang II”, and “shock”, or “vasoplegia”, or “vasodilatory shock”, or “vasoplegic shock”.

The study was registered at the International Prospective Register of Systematic Reviews (PROSPERO): CRD42024510254.

### 2.1. Study Selection and Data Extraction

The titles and abstracts of the identified records were screened by two independent review authors (AA/HM or BA/AS). Records deemed irrelevant according to our eligibility criteria were excluded. All relevant records were retrieved in full text and assessed for inclusion in the qualitative and quantitative analyses. Disagreements were resolved through discussions among the reviewers until a consensus was reached. The included articles were tabulated with the following data: study’s last author; year of publication; study design; study population; intervention and comparator arms; demographics of the study population, including age and gender; background vasopressor(s); cause of shock requiring angiotensin II use; follow up duration; and clinical outcomes reported.

### 2.2. Outcomes

The primary outcome was in-hospital mortality associated with angiotensin II administration compared to standard therapy, while the secondary outcomes included MAP change with angiotensin II, multi-organ failure (MOF), and the incidence of atrial fibrillation (AF).

### 2.3. Assessment of Quality and Risk of Bias

The risk of bias in the included studies was evaluated by two independent review authors (AR/AAA). The Cochrane Collaboration’s tool and the Risk of Bias In Non-randomized Studies of Interventions (ROBINS-I) assessment tool were used for RCTs and observational studies, respectively [12,13]. Disagreements were resolved through discussions and consensus. For RCTs, five main bias risks were evaluated, including selection bias, performance bias, detection bias, attrition bias, reporting bias, and other risks as identified by the two reviewers assessing the risk of bias. Using these key domains, the overall risk of bias was adjudicated as low if all key domains had a low risk of bias, as high if at least one domain was evaluated to carry a high risk of bias, or as unclear overall risk of bias if the risk of bias was found to be low or unclear for all domains. For observational studies, the ROBINS-I tool consists of seven domains, and the overall risk of bias in each study was judged to be low if the risk of bias was found to be low in all domains, moderate if the risk of bias was evaluated as low or moderate in all domains, serious if the risk of bias was serious in at least one domain, critical if the risk of bias was critical in at least one domain, and no information when there was lack of information in one or more domains given that the study was not at serious or critical risk of bias in any of the seven domains.

The certainty of evidence for each outcome was assessed by two review authors (AR/AAA) using the Grading of Recommendations Assessment, Development, and Evaluation (GRADE) tool, ranging from very low to high overall quality according to the judgment of the risk of bias, inconsistency, indirectness, imprecision, and publication bias.

### 2.4. Data Analysis

Using the random effects model (Mantel–Haenszel), data were combined in forest plots for the outcomes of interest, including in-hospital mortality, MAP change, MOF, and AF, and the results were reported as risk ratio (RR) with 95% confidence intervals (CI), and two-tailed *p*-value < 0.05 indicated statistical significance. The eterogeneity of data was assessed by the Q test and I^2^, with I^2^ > 50% denoting marked heterogeneity. In view of the small number of identified studies, publication bias could not be examined by visual inspection of the funnel plots or the Egger test, as the required criteria for the test were not met [14]. Meta-analyses were performed using Review Manager software (version 5.4; The Cochrane Collaboration, Software Update, Oxford, UK).

## 3. Results

### 3.1. Included Studies

**Figure 1:**
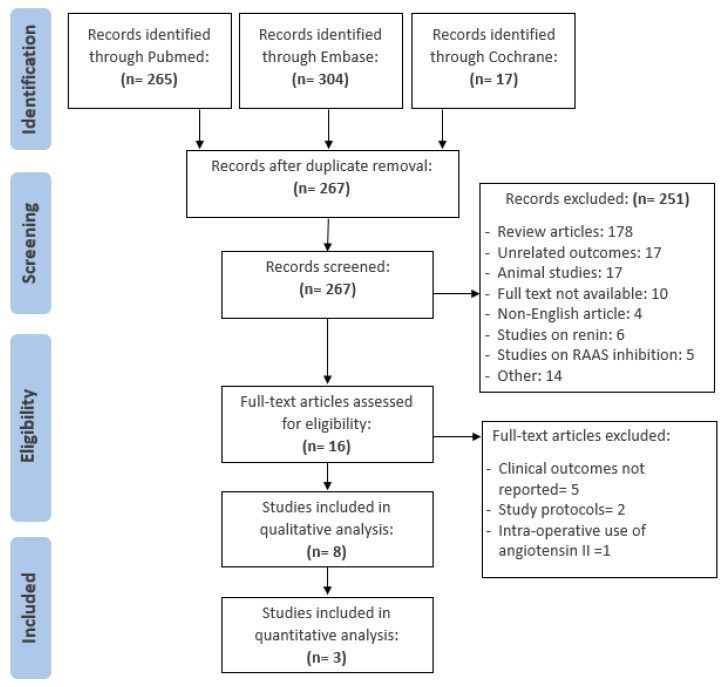
The PRISMA Flow Diagram.

A total of 586 records were identified in the initial literature search using PubMed, Embase, and Cochrane Database. After removing duplicate records, 267 records were screened, of which 251 records were excluded due to variable reasons as demonstrated in Figure 1. A total of sixteen articles were evaluated in full text for eligibility, of which eight were excluded due to the following reasons: non-clinical outcomes (*n* = 5), study protocol only (*n* = 2), and intra-operative use of angiotensin II. Eventually, a total of eight studies (*n* = 974 patients) evaluating the use of angiotensin II in vasoplegic shock were included in the qualitative analysis and only three studies comprising 461 patients were included in the quantitative analysis. The key characteristics of the included studies (*n* = 8) are summarized in Table 1. Among the studies included, two studies were RCTs, one study was a post hoc analysis of the landmark ATHOS-3 trial, four were retrospective observational studies, and one study was a prospective observational study.

Angiotensin II versus placebo for catecholamine-refractory vasodilatory shock was evaluated in the ATHOS-3 trial, the post hoc analysis of ATHOS-3 by Klijian and colleagues for angiotensin II in vasoplegia after cardiac surgery with cardiopulmonary bypass (CBP), and in a single-center RCT by Chawla et al. for high output shock, and was shown to have a similar impact on 30-day in-hospital mortality (Khanna et al. 46% vs. 54%; *p* = 0.12, Klijian et al. 11% vs. 14%; *p* > 0.05, Chawla et al. 50% vs. 60%; *p* = 1), while MAP increase was significant with angiotensin II use, as demonstrated in Table 1 [9,15,16]. In another approach, Wieruszewski et al. [17] assessed the responsiveness to angiotensin II in a multicenter retrospective study of 270 patients and found that responsiveness to angiotensin II was associated with reduced mortality (HR: 0.50; 95% CI, 0.35–0.71; *p* < 0.001). Interestingly, Wieruszewski and colleagues evaluated the impact of angiotensin II among 50 patients with mechanical circulatory support (MCS) and demonstrated that among patients with cardiac failure (*n* = 41), angiotensin II administration was associated with 54% 28-day mortality, despite its significant impact on MAP increase [18]. As a primary vasopressor in a prospective observational study, angiotensin II compared to conventional vasopressor therapy in vasodilatory shock demonstrated similar mortality at 28 days (18% vs. 29%; *p* = 0.18) [19].

**Table 1:** Summary of Studies.

Author, Year	Design, Country, Follow-Up	Population (Inclusion Criteria)	Intervention and Control	Age (Years)	Gender (Male)	Primary Outcome	Background Vasopressor	Shock Cause	Outcomes
**Khanna et al., 2017 [9]**	RCT in 9 countries (USA, Australasia, and Europe) Follow-up: 28 days	- Age ≥ 18 years - Vasodilatory shock despite IV volume resuscitation over 24 h and high dose vasopressors (NA: 0.2 mcg/kg/min or equivalent dose of another vasopressor)	Ang II (*n* = 163) vs. saline placebo (*n* = 158)	Ang II: 63 (52–75) * Placebo: 65 (53–75) *	Ang II: 56% Placebo: 65%	MAP response at 3 h defined as increase from baseline 10 mmHg or increase to 75 mmHg, without increase in background vasopressor dose	NE equivalent dose: * Ang II: 0.33 (0.23–0.56) mcg/kg/min Placebo: 0.34 (0.23–0.56) mcg/kg/min	**Ang II:** Sepsis: 78% Postoperative vasoplegia: 6% Multifactorial: 4% Other, potentially sepsis: 12% Placebo: Sepsis: 84% Postoperative vasoplegia: 6% Multifactorial: 3% Other, potentially sepsis: 7% Pancreatitis: 1%	**In-Hospital mortality:** 46% vs. 54%; HR 0.78 (0.57–1.07); *p*-value 0.12 **MAP change at 3 h:** 67% vs. 23%; OR 7.95 (4.76–13.3); *p*-value < 0.001 **Multiorgan failure:** 15.3% vs. 14.6% **AF:** 3.1% vs. 3.2%
**Wieruszewski et al., 2021 [17]**	Retrospective study (5 centers), USA) Follow-up: 30 days	- Age ≥ 18 years - Shock treated with ang II	Ang II (*n* = 270) Then characteristics of responders (*n* = 180) and non-responders (*n* = 89) were compared	Responders: 61 ± 14 Non-responders: 57 ± 17	Responders: 68% Non-responders: 62%	Responsiveness to ang II defined as attainment of MAP > 65 mm Hg with a stable or reduced total vasopressor dosage within 3 h	NE equivalent-dose: Responders: 0.57 ± 0.33 mcg/kg/min Non-responders: 0.55 ± 0.35 mcg/kg/min	Responders: Sepsis: 57% Postoperative vasoplegia: 10% Hemorhagic: 4% Obstructive: 2% Multifactorial 27% Non-responders: Sepsis: 52% Postoperative vasoplegia: 11% Hemorhagic: 5% Obstructive: 5% Multifactorial 28%	Responsiveness to ang II: 67% **In-hospital mortality:** Responsiveness to ang II was associated with reduced mortality: HR: 0.50; 95% CI, 0.35–0.71; *p* < 0.001
**Klijian et al., 2021 [15]**	Post hoc analysis of ATHOS-3 Follow-up: 28 days	- Age ≥ 18 years - Vasoplegia after cardiac surgery with CPB	Ang II (*n* = 9) vs. Placebo (*n* = 7)	Ang II: <65 y: 5 (55.6) >65 y: 4 (44.4) Placebo: <65 y: 2 (28.6) >65 y: 5 (71.4)	Ang II: 88% Placebo: 71%	MAP response at 3 h defined as increase from baseline 10 mmHg or increase to 75 mmHg, without increase in background vasopressor dose	NE equivalent dose: * Ang II: 0.28 (0.20–0.47) Placebo: 0.29 (0.23–0.40)	Vasoplegia after cardiac surgery: 100%	**MAP change at 3 h:** 88.9% vs. 0; *p* = 0.0021 **In-hospital mortality:** 11% vs. 14%; *p*-value > 0.05
**Smith et al., 2022 [20]**	Retrospective study (5 centers), USA Follow-up: 28 days	- Age ≥ 18 years - All shock states treated with ang II	Ang II (*n* = 162) **	63 (51–71) *	58.6%	MAP and norepinephrine equivalent dose difference between hour 0 and hour 3 of post-ang II	Baseline NE-equivalent dose: * 0.55 (0.38–0.82)	Septic shock: 72.2% Cardiogenic shock: 21% Non-septic distributive shock: 14.2% Hypovolemic shock: 3.1%	MAP change at 3 h: * 62 (57–70) vs. 73 (65–79); difference 9.3 (6.4–12.1); *p*-value < 0.001 NE equivalent dose change at 3 h: * 0.55 (0.38–0.82) vs. 0.39 (0.16–0.64); difference 0.16 (0.10–0.22); *p*-value < 0.001
**Zangrillo et al., 2020 [21]**	Retrospective study (1 center), Italy Follow-up: 48 h	- Ventilated patients with COVID-19-associated vasodilatory shock	Ang II: 14 ** ¶	64 (54–69) *	73.3%	Change in oxygenation parameters and MAP within 48 h of ang II	10 used ang III as first agent, and 4 as second-line agent	COVID-19 infection-associated vasodilatory shock	**PaO2/FiO2:** * 121.4 (98.1–218.1) vs. 200.0 (168.0–248.5) MAP change: * 71 (65–79) vs. 85 (80–87)
**Chawla et al., 2014 [16]**	RCT (1 center), USA Follow-up: 30 days	- Age ≥ 21 years - High output shock, which was defined as SOFA score of 4 and cardiac index > 2.4 L/min/BSA 1.73 m^2^.	Ang II (*n* = 10) Placebo (*n* = 10)	Ang II: 68 ± 17 Placebo: 57 ± 12	Ang II: 60% Placebo: 90%	NE dose to maintain MAP of 65 mmHg	Ang II: NE: 19.80 ± 11.67 mcg/min Vasopressin: 0.02 ± 0.03 unit/min Placebo: NE: 30.30 ± 20.37 mcg/min Vasopressin: 0.05 ± 0.02 unit/min	High output shock	**In-hospital mortality:** 50% vs. 60%; *p*-value 1 **MAP change at 3 h:** 73.1 ± 12.5 vs. 68.8 ± 7.0 **Multiorgan failure:** 20% vs. 30%; *p*-value 1 **AF:** 20% vs. 0; *p*-value 0.47
**Wieruszewski et al., 2023 [18]**	Retrospective study (4 centers), USA Follow-up: 28 days	- Age ≥ 18 years - MCS device (ECMO, LVAD, Impella^®^, TandemHeart^®^) at ang II use	Ang II (*n* = 50) *Divided:* *Cardiac failure (n = 41)* *Respiratory failure (n = 9)*	Cardiac failure: * 62 (47–71) Respiratory failure: * 49 (38–64)	Cardiac failure: 88% Respiratory failure: 78%	- MAP and NE-equivalent dose change within 12 h of ang II - Mortality	NE-equivalent dose: * Cardiac failure: 0.44 (0.34–0.64) Respiratory failure: 0.47 (0.33–0.73)	Sepsis shock: Cardiac failure: 17% Respiratory failure: 78% Cardiogenic shock: Cardiac failure: 34% Respiratory failure: 0 Vasoplegic shock: Cardiac failure: 73% Respiratory failure: 11% Hemorrhagic shock: Cardiac failure: 17% Respiratory failure: 0 Obstructive shock: Cardiac failure: 7% Respiratory failure: 0 Others: Cardiac failure: 5% Respiratory failure: 11%	MAP change: * Cardiac failure: 60 (57–73) vs. 70 (67–85); *p*-value < 0.001 Respiratory failure: 61 (57–72) vs. 81 (70–84); *p*-value 0.26 **NE-equivalent dose change:** Cardiac failure: Declined by 0.16 mcg/kg/min; *p*-value < 0.001 Respiratory failure: Decline by 0.13 mcg/kg/min; *p*-value 0.06 **Mortality at 28 days:** Cardiac failure: 54% Respiratory failure: 22%
**See et al., 2023 [19]**	Prospective observational study (1 center), Australia Follow-up: 90 days	- Age ≥ 18 years - Vasodilatory hypotension, received vasopressor(s) for <24 h	Ang II as primary vasopressor (*n* = 40) vs. Control: conventional vasopressors (*n* = 80)	Ang II: 62 ± 12 Control: 63 ± 14	Ang II: 70% Control: 64%	- Peak serum creatinine level at 7 days - Mortality	Ang II: * NE: 12 (7–18) mcg/min Vasopressin: 0.04 (0.02–0.04) unit/min Epinephrine: 5 (5–5) mcg/min Control: * NE: 10 (5–20) mcg/min Vasopressin: 0.04 (0.04–0.04) unit/min Epinephrine 5 (4–9) mcg/min	Sepsis: Ang II: 60% Control: 55% Pancreatitis: Ang II: 5% Control: 0 Post-operative: Ang II: 15% Control: 16% Multifactorial: Ang II: 10% Control: 11% Others: Ang II: 10% Control: 18%	**Peak creatinine at day 7:** * 128 (89–192) vs. 126 (91–174); *p*-value = 0.81 **Mortality at 28 days:** 18% vs. 29%; *p*-value = 0.18 **Mortality at 90 days:** 22% vs. 30%; *p*-value = 0.39

* Median (IQR); ** single-arm study without a comparator control; ¶ one was excluded due to unobtainable data; **CPB:** cardiopulmonary bypass; **NE:** norepinephrine; **SOFA:** sequential organ function assessment; **MCS:** mechanical circulatory support; **AKI:** acute kidney injury; Ang II: angiotensin II.

### 3.2. Risk of Bias Assessment

**Figure 2:**
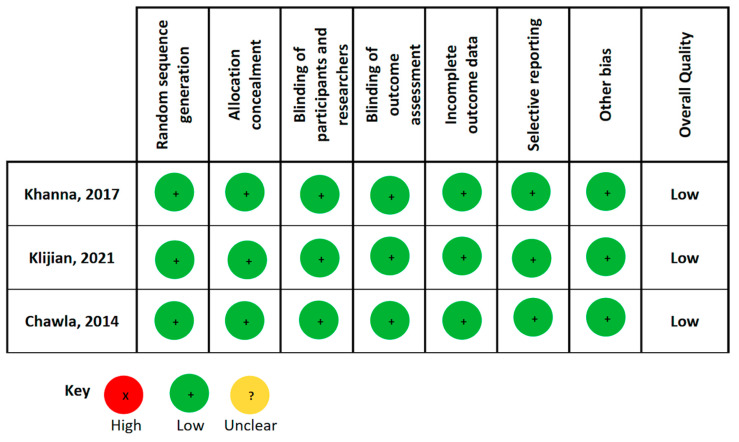
Risk of bias assessment [9,15,16].

**Figure 3:**
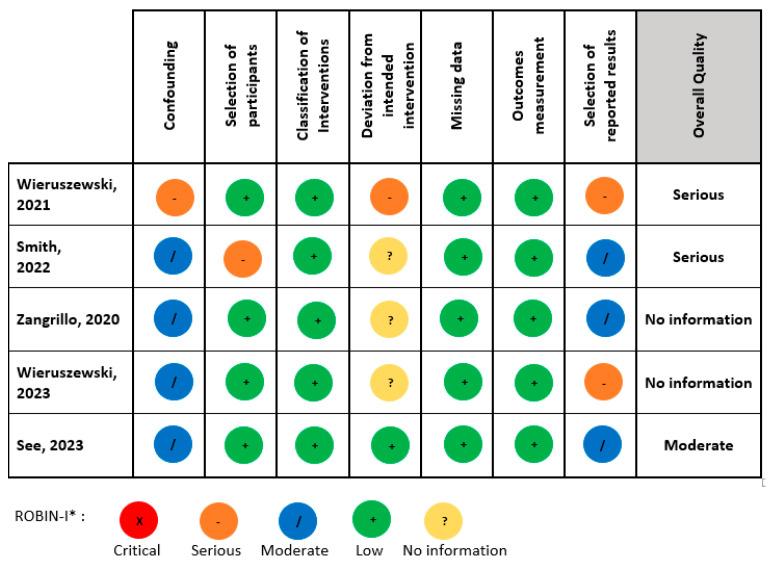
Risk of bias assessment for included non-randomized studies using ROBIN-I* quality assessment tool [17,18,19,20,21].

Figure 2 demonstrates the results of risk of bias assessments for included RCTs using Cochrane Collaboration’s tool. All included RCTs were evaluated by two independent review authors to have a low risk of bias. Nevertheless, the five observational studies included demonstrated different levels of bias as evaluated by the ROBIN-I* quality assessment tool. Two studies were judged to have an overall serious bias risk, one study a moderate bias risk, and two studies as no information (Figure 3).

### 3.3. Clinical Outcomes

#### 3.3.1. In-Hospital Mortality

**Figure 4:**
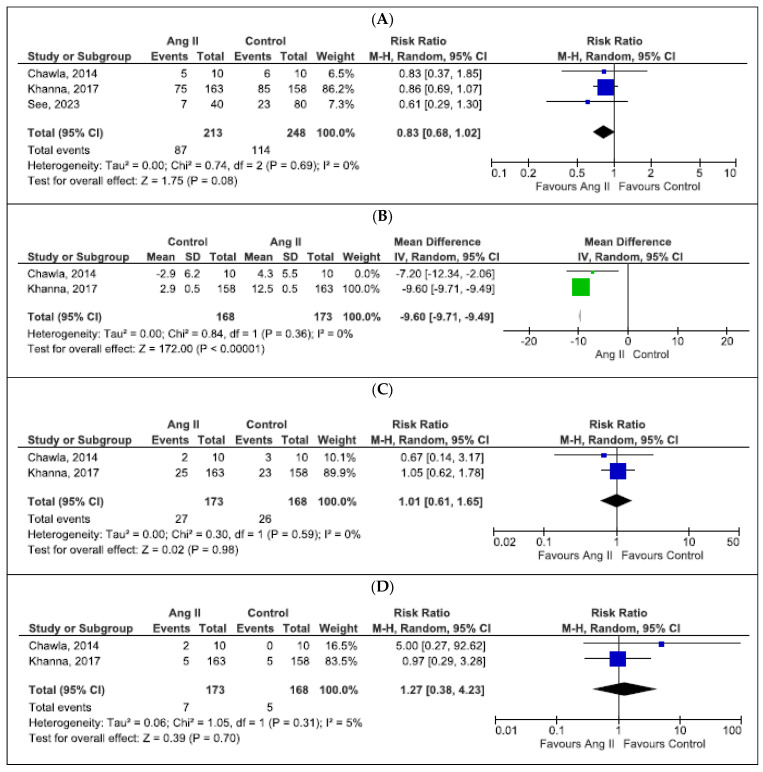
Forest plots of clinical outcomes of angiotensin II use [9,16,19]. (**A**) Morality, (**B**) MAP change, (**C**) Multiorgan failure, (**D**) Atrial fibrillation.

Three of the eight studies reported in-hospital mortality with angiotensin II use (*n* = 461) and were hence included in the meta-analysis with 213 patients receiving angiotensin II and 248 patients in the control arm. Overall, angiotensin II use demonstrated similar in-hospital mortality compared to standard therapy (RR = 0.83; 95% CI, 0.68–1.02, I^2^ = 0%), as presented in Figure 4A.

#### 3.3.2. MAP Change

Two studies (*n* = 341) reported MAP change within three hours of angiotensin II initiation compared to placebo and demonstrated significant MAP increase with angiotensin II use (mean difference = −9.60; 95% CI, −9.71, −9.49, I^2^ = 0%), as presented in Figure 4B. Studies reporting MAP change before and after angiotensin II use without a comparator arm were not included in this analysis [18,20,21].

#### 3.3.3. Safety Outcomes

As demonstrated in Figure 4C,D, there was no difference in MOF and AF with angiotensin II use compared to standard therapy (MOF: RR = 1.01; 95% CI, 0.61–1.65, I^2^ = 0%; AF: RR = 1.27; 95% CI, 0.38–4.23, I^2^ = 5%).

### 3.4. Certainty of Evidence Assessment

The certainty of evidence of the outcomes assessed using the GRADE tool revealed that in-hospital mortality, MOF, and AF had moderate certainty, while MAP change had high certainty, as demonstrated in Table 2.

## 4. Discussion

The present study demonstrates that the administration of angiotensin II in vasodilatory shock was not associated with a significant decrease in all-cause mortality in comparison to standard therapy. In addition, our analysis revealed no difference in the occurrence of MOF or AF; nevertheless, we observed a statistically significant increase in the MAP in those who received angiotensin II.

The renin–angiotensin system is a complex network of crucial importance in maintaining arterial pressure, blood volume, and sodium balance. Although it has traditionally been considered a circulating system, a number of recent works underline the “local RAS” in organs, contributing to the synthesis of angiotensin II. Angiotensin II plays a vital role in vasoconstriction and stress responses.

The naturally occurring angiotensin II is produced via the activation of the renin–angiotensin–aldosterone system. The latter is a complex system comprising several enzymes and proteins. Renin is produced by the juxtaglomerular cells of the kidneys in response to decreased renal blood flow and sympathetic stimulation. Renin is essential for the formation of angiotensin II through the activation of angiotensinogen production in the liver followed by the formation of angiotensin I by the angiotensin-converting enzyme in the lungs prior to the result formation, which is angiotensin II [22,23].

Catecholamines remain the first-line vasopressors in the treatment of sepsis, but their numerous side effects on the cardiovascular system, including tachycardia, arrhythmias, and metabolic disturbances, are particularly dangerous when administered at high doses. This is generating an interest in catecholamine-sparing strategies, where angiotensin II could be used to provide comparable hemodynamic findings but with reduced complications. Angiotensin II has vasoconstrictive effects and supports sympathetic outflow, thus providing an appropriate alternative in cases when adrenergic agents are not sufficient.

Current guidelines recommend NE as a first-line vasopressor for the management of sepsis and septic shock, and vasopressin to be considered a second-line agent in catecholamine resistance shock [24,25]. In the ATHOS-3 trial, the addition of angiotensin II to NE resulted in augmented MA [9]. In the state of cardiogenic shock, in particular, NE has an established ground for use in patients with systolic BP less than 90 mmHg as the vasopressor of choice with additional inotropic support.

Wieruszewski et al. reported that in patients with cardiogenic shock, angiotensin II administration resulted in hemodynamic improvement, higher MAP, and fewer vasopressor requirements [18]. In an animal study, Garcia et al. speculated that angiotensin II— in addition to immediate effects on hemodynamics—may possess additional biological functions [26]. Emerging literature suggests an inhibitory role in inflammation, which may help to curb the deleterious consequences of exaggerated inflammation response typically encountered in vasoplegic shock. The inflammatory modulation of the myocardium in septic shock was associated with higher myocardial inflammatory molecule secretion in NE treatment than in treatment with angiotensin II. In particular, the myocyte mRNA generation of interleukin-1, interleukin-6, and its receptor through that mechanism further reduces the inflammatory-induced increase in myocardial oxygen demand. Additionally, in other types of shock-associated vasoplegia/distributive shock, such as spinal shock, NE is still considered the first-line medication; this could be a potential field for the future application of angiotensin II [27,28]. In cases of catecholamine-resistant vasodilatory shock, especially of septic origin, patients tend to have high renin and angiotensin I but low angiotensin II, with a correspondingly poor prognosis. Contributing factors are increased angiotensinase activity and angiotensin II degradation, leading to impaired signaling and reduced vasoconstriction. These physiological abnormalities could lead to poor outcomes in sepsis and, as such, constitute an indication of therapies that intervene along this pathway [23].

Our meta-analysis has demonstrated a favorable hemodynamic effect of angiotensin II by augmenting the MAP, which is an established target in shock management, without reducing all-cause mortality. This could be attributed to the short follow-up duration in the majority of the included studies which evaluated mortality at one month. This short-term follow-up has been disputed in a large review of 343 RCTs by Friedrich et al., which reported that mortality effects persisted for 60 to 90 days in critically ill patients following recruitment [29]. The effects of angiotensin II on MAP increase and the restoration of circulation should be considered for different clinical outcomes: firstly, the direct effect of angiotensin II on the hemodynamics and MAP; secondly, adding angiotensin II to the management might help reduce NE requirements and mitigate the need to add a third agent such as epinephrine, which further protects against the undesirable effects of high dosage NE or of a potent catecholamine medication (such as epinephrine) directly [27,28].

In a recently published expert consensus by the Italian Society of Anesthesia Analgesia Reanimation and Intensive Care (SIAARTI) for the use of angiotensin II in the management of distributive shock, four main domains were highlighted concerning angiotensin II utilization, the knowledge gap, benefits, physiological advantages, and barriers to using it. The challenging knowledge gap in terms of refractory shock definition, the timing of non-catecholaminergic pressors initiation, and the de-escalation approach of angiotensin II remain critical concerns to be addressed. The consensus suggested that angiotensin II might be useful among patients with vasoplegic shock despite high vasopressor requirements, and among patients with renal impairment requiring renal replacement therapy in the setting of septic shock necessitating high vasopressor doses [30].

Our report has a number of significant limitations. First, the small number of studies included and patients recruited are such. Second, publication bias could not be assessed given the small number of studies fulfilling the eligibility criteria of the meta-analysis, which limited the utilization of funnel plots or Egger’s test. Third, meanwhile, the benefits of angiotensin II may extend to patients with acute kidney injury or those with high-renin levels, renal outcomes of angiotensin II could not be evaluated in our review as the renal outcomes were not reported in almost all the included studies, warranting future research to address the renal outcomes of angiotensin II. However, this meta-analysis demonstrated that angiotensin II is a promising vasopressor gaining a great interest in the field of critical care research. Nevertheless, its use as a primary vasopressor agent in vasodilatory shock, either due to sepsis or post-cardiopulmonary bypass needs to be further evaluated in large RCTs.

## 5. Conclusions

In vasodilatory shock, angiotensin II use demonstrated comparable in-hospital mortality compared to standard therapy. Nevertheless, it resulted in significant MAP changes, which may encourage clinicians to use it in cases of profound hypotension.

## Figures and Tables

**Table 2:** Certainty of evidence of outcomes.

Outcome	Studies	Risk of Bias	Inconsistency	Indirectness	Imprecision	Publication Bias	Overall
**Mortality**	2 RCTs, 1 observational	Not serious	Not serious	Not serious	Serious * (−1)	Not serious **	⊕⊕⊕⊘
**MAP Change**	2 RCTs	Not serious	Not serious	Not serious	Not serious	Not serious **	⊕⊕⊕⊕
**Multi-Organ Failure**	2 RCTs	Not serious	Not serious	Not serious	Serious * (−1)	Not serious **	⊕⊕⊕⊘
**Atrial Fibrillation**	2 RCTs	Not serious	Not serious	Not serious	Serious * (−1)	Not serious **	⊕⊕⊕⊘

* The outcome of interest was a secondary outcome; ** the number of studies made it difficult to judge accurately; ⊕⊕⊕⊕ represents high certainty, ⊕⊕⊕⊘ represents moderate certainty.

## Data Availability

Data used in this meta-analysis are available on request from the corresponding author.

## Supplementary Materials

The following supporting information can be downloaded at: https://www.mdpi.com/article/10.3390/life14091085/s1, PRISMA checklist for the current meta-analysis. Ref. [31] is cited in the Supplementary Materials.
